# The role of children in the transmission of SARS-CoV2: updated rapid review

**DOI:** 10.7189/jogh.10.021101

**Published:** 2020-12

**Authors:** Xue Li, Wei Xu, Marshall Dozier, Yazhou He, Amir Kirolos, Zhongyu Lang, Peige Song, Evropi Theodoratou, on behalf of UNCOVER

**Affiliations:** 1 School of Public Health and the Second Affiliated Hospital, Zhejiang University, Hangzhou, China; 2 Centre for Global Health Research, Usher Institute, University of Edinburgh, Edinburgh, United Kingdom; 3 Information Services, University of Edinburgh, Edinburgh, United Kingdom; 4 Department of Clinical Infection, Microbiology & Immunology, Institute of Infection, Veterinary & Ecological Sciences, University of Liverpool, Liverpool, United Kingdom; 5 Cancer Research UK Edinburgh Centre, Medical Research Council Institute of Genetics and Molecular Medicine, University of Edinburgh, Edinburgh, United Kingdom

## Abstract

**Background:**

Understanding carriage and transmission potential of SARS-CoV-2 in children is of paramount importance to understand the spread of virus in school and community settings.

**Methods:**

We performed an updated rapid review to investigate the role of children in the transmission of SARS-CoV-2. We synthesized evidence for five categories and results are reported narratively.

**Results:**

A total of 33 new studies were included for this review. We did not identify additional studies that reported documented cases of SARS-CoV-2 transmission by children. We identified 15 new studies that demonstrate children’s susceptibility and transmission risk of SARS-CoV-2 with evidence provided on the chance of being index or secondary cases, the potential of faecal-oral transmission, and the possibility of asymptomatic transmission. There is little data on the transmission of SARS-CoV-2 in schools. There were three studies reporting COVID-19 school outbreaks in France (Oise), Australia (New South Wales) and Israel. The remaining four studies found that all reported cases did not infect any other pupils or staff. With data from seven studies and governmental websites, the proportion of children among all confirmed COVID-19 patients was estimated for 29 countries, varying from 0.3% (lowest in Spain) up to 13.8% (highest in Argentina). Lastly, we identified seven studies reporting on PIMS-TS linked to COVID-19 among paediatric patients.

**Conclusions:**

There is somewhat limited evidence available for quantifying the extent to which children may contribute to overall transmission, but the balance of evidence so far suggests that children and schools play only a limited role in overall transmission.

The outbreak of coronavirus disease 2019 (COVID-19), caused by the infection of severe acute respiratory syndrome coronavirus 2 (SARS-CoV-2), poses a global health, societal and economic threat. Understanding carriage and transmission potential of SARS-CoV-2 in children is of paramount importance to prevent the spread of the virus in school and community settings.

Initial evidence suggests children may be less frequently infected than adults, and typically tend to have mild symptoms when infected. Emerging reports of a very rare Paediatric Inflammatory Multisystem Syndrome Temporally associated with SARS-CoV-2 (PIMS-TS) suggest a link to COVID-19 in paediatric patients [1]. Although we have learned much about COVID-19 since the outbreak, the major question on the extent children contribute to SARS-CoV-2 transmission remains unanswered. Understanding this issue is vital to inform decisions about re-opening schools/childcare facilities, which could have strong implications for children’s physical well-being, mental health and learning [2].

Our early rapid review of transmission in children identified several relevant studies and provided useful but limited information on susceptibility and transmission in children. We have now updated our rapid review and included large seroprevalence surveys, national or municipal population screening studies and tracing studies on school-based clusters of transmission. We aim to keep updating the rapid review to include new studies as they become available and to re-evaluate the conclusions given the rapid pace of ongoing research on COVID-19.

## METHODS

### Literature search and eligibility criteria

We searched PubMed, medRxiv and the WHO COVID-19 database on 21 June 2020 with entry date limits from late 2019 (please see search strategies in Appendix S1 of **the** **Online Supplementary Document**), to identify studies that investigated transmission of SARS-CoV-2 in children or in schools. We reviewed titles, abstracts, and subsequently full texts to identify eligible articles based on a set of predefined inclusion and exclusion criteria. We hand-searched reference lists of the retrieved eligible publications to identify any additional relevant studies. In particular, we included 1) studies reporting documented COVID-19 cases transmitted by SARS-CoV-2 positive children; 2) studies presenting indirect evidence on the potential of SARS-CoV-2 transmission by (both symptomatic and asymptomatic) children; 3) studies reporting cluster outbreaks of COVID-19 in schools; 4) studies estimating the proportions of children infected by SARS-CoV-2 and the presence of PIMS-TS. We also included data on COVID-19 statistics from governmental websites or reports. Conversely, we excluded studies investigating clinical features and/or treatment of paediatric COVID-19 cases without any information on transmission. We included articles in peer-reviewed journals and pre-prints and excluded comments, conference abstracts, and interviews. We restricted studies to those reported in English or Chinese. In addition, we summarized and checked the references of previous reviews and policy briefs on the transmission of SARS-CoV-2 among children.

### Data extraction and evidence synthesis

Data relevant to the evidence for transmission of SARS-CoV-2 by children were extracted by five reviewers (XL, WX, YH, AK, ZL) and checked by a senior epidemiologist (ET). We synthesized evidence thematically and reported results narratively.

## RESULTS

**Figure 1:**
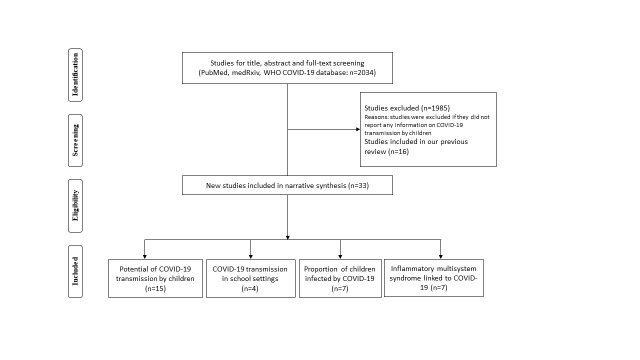
Flowchart summarizing study identification and selection.

The initial search retrieved 2034 titles of articles. After screening, 33 new studies were eligible for inclusion since our last search on 30 April 2020 (**Figure 1**). Findings for the previously included studies have been summarised in our early rapid review [3]. For this update, we did not identify additional studies that reported documented cases of SARS-CoV-2 transmission by children; we identified 15 new studies presenting indirect evidence on the potential of child transmission [2,4-17], four new studies exploring school/d-care outbreaks or tracing close contacts of SARS-CoV-2 transmission in school settings [18-21], and seven studies reporting on PIMS-TS [22-28]. We also found seven new studies [9,29-34] and obtained data from governmental websites for 29 countries that provided estimation on the proportions of children infected by SARS-CoV-2.

### Summary of findings from previous rapid review

There is limited evidence detailing transmission of SARS-CoV-2 from infected children based on our previous review (covering the period up to 30 April 2020) [3]. We found two studies that reported a 3-month old infant, whose parents developed symptomatic COVID-19 seven days after caring for the infant [35] and two children who may have contracted COVID-19 from the initial cases at a school in New South Wales [36]. In addition, we identified six studies presenting indirect evidence on the potential for SARS-CoV-2 transmission by children [37-42], three of which found prolonged virus shedding in stools. There is little data on the transmission of SARS-CoV-2 in schools. We identified only two studies reporting outbreaks of COVID-19 in school settings and one case report of a child attending classes but not infecting any other pupils or staff [36,43,44]. Data from population-based studies in Iceland, Italy, South Korea, Netherlands, California and a hospital-based study in the UK suggest children may be less likely to be infected. Overall, there is very limited evidence on paediatric cases acting as a source of infection, which highlights the importance of obtaining robust data on transmission dynamics in children in future studies.

### Susceptibility and transmission risk of SARS-CoV-2 in children

From this update, we identified 15 new studies that demonstrated the children’s susceptibility and transmission risk of SARS-CoV-2 with evidence provided on the chance of being index and secondary cases, the potential of faeco-oral transmission, and the possibility of asymptomatic transmission.

Contact-tracing studies suggested that children were not likely to be the index case in households and children contacts were less likely to be secondary cases than adult contacts. A collection of international family clusters found that children were the index case in only 3 (10%) of 31 individual cluster studies [4]. Data from Guangzhou have supported this, reporting an even lower rate (5%) of children as index cases in households [5]. A family-based study involving 239 participants from 185 housemates in the Netherlands found no indications in any of the family clusters that a child was the source of COVID-19 transmission [6]. There are at least 8 studies suggesting a lower secondary attack rate for children than their adult counterparts [5,7-13]. Meta-analysis of these studies showed that the pooled odds ratio (OR) of a child being an infected contact was 0.44 (95% confidence interval (CI) = 0.29-0.69), suggesting differential susceptibility between children and adults [14].

Persistent shedding of SARS-CoV-2 in stools of infected children, which raised the concern of faeco-oral transmission, is supported by a new study. Hua *et al* conducted a retrospective multi-centre study, and followed up all children from 883 families with SARS-CoV-2 infected members in Zhejiang Province, China [15]. They found that faecal SARS-CoV-2 RNA detection was positive in 91.4% (32/35) cases and about 33.3% of paediatric patients persisted with faecal shedding for 14 days after hospital discharge. However, they observed no subsequent infection in family contacts of faecal viral excreting children.

The proportion of children who are truly asymptomatic or pre-symptomatic remains unknown. Data from Italian emergency departments suggested that 21% of SARS-CoV-2 PCR-positive children were asymptomatic at the time of testing, but there was no follow-up to determine whether these children developed symptoms later (pre-symptomatic) [2]. This figure is considerably higher than the 4% reported by a nationwide case-series of 2135 paediatric patients in China [37]. However, it is likely that the true rate of asymptomatic infection was underestimated by the hospital-based study in China since many asymptomatic children are unlikely to be hospitalised or tested. In any case, it is thought that presymptomatic transmission plays a role in the overall spread of COVID-19 [15,37].

A hospital-based study in South Korea examined the viral load of 12 infected children and demonstrated that symptomatic children had higher initial viral load in nasopharyngeal swab specimens than asymptomatic children, but found no differences in faeces or saliva specimens [16]. Additionally, another study of 35 paediatric patients reported that the median SARS CoV-2 viral load was higher in symptomatic than asymptomatic paediatric patients, and patients <5 years had higher viral loads and were more likely to be symptomatic than patients ≥5 years old [17].

### SARS-CoV-2 transmission in school settings

Four new studies on SARS-CoV-2 transmission in school settings were identified. A cross-sectional study in Belgium examined the transmission risk of SARS-CoV-2 in day-care settings. They randomly sampled 84 children who were attending day-care after the outbreak of COVID-19, and found no asymptomatic carriage of SARS-CoV-2 among young children [18]. Another school-based tracing study in Ireland examined all reported paediatric cases of COVID-19 attending school during the pre-symptomatic and symptomatic periods of infection (n = 3) and did not identify any cases of onward transmission to other children or adults within the school and a variety of other settings [19]. Nationwide surveillance in Singapore identified two SARS-CoV-2 positive students who attended their respective schools on the first day of their symptoms before subsequently being diagnosed with COVID-19 [20]. Screening of students and staff who were close contacts did not detect any SARS-CoV-2 infection. Reports from 25 municipal public health services (GGDs) in the Netherlands found no possible COVID-19 clusters that had a link to schools or childcare facilities before the schools closed on 16 March. After reopening the primary schools and childcare facilities, a few infections among employees at schools were reported (up to early June) by GGDs, but children in school settings were not found to be the index cases. Although most countries did not observe a significant increase in COVID-19 cases after schools reopened, cases in Israel more than doubled within 50 days after schools opened. The increase in cases has largely been associated with children between the ages of 10-19 years [21]. This should be interpreted with caution as other factors (eg, general reopening of other aspects of society) may have also contributed and the rise in cases is unlikely to be solely due to schools reopening given that it is a temporal association.

### Proportions of children infected by SARS-CoV-2

The very large Spanish ENE-COVID seroprevalence survey (n = 61 075) conducted between 27/04/2020-11/05/2020 found lower seroprevalence in children (n = 6527) [45]. Specifically, the seroprevalence of COVID-19 was estimated to be 3.1% (95% CI = 2.2%-4.2%) in children aged 5-9 years, 4.0% (95% CI = 3.1%-5.0%) in those aged 10-14 years, and 3.7% (95% CI = 2.9%-4.8%) for those aged 15-19 years, comparing with a seroprevalence estimate of 5.0% (95% CI = 4.7%-5.4%) in overall population. Another large seroprevalence survey in Sweden (conducted by the end of April) found that 4.9% of those aged 0-19 tested positive for SARS-CoV-2 antibodies, compared to 6.7% of those aged 20-64 [46].

**Figure 2:**
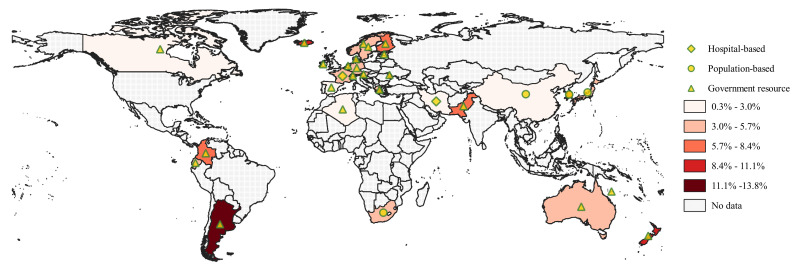
The proportion of children among the confirmed COVID-19 patients (data available for 29 countries).

International research confirms that the percentage of children among the confirmed COVID-19 patients is low. A study analysed the global COVID-19 prevalence for 23 countries with data available for paediatric cases [47]. It was reported that about 1.9% (8113 out of 424 978) of confirmed COVID-19 cases were children; the admission rate was 3.9% and the ICU admission rate was 0.3% among paediatric patients. Another study examined age-specific COVID-19 data which had been collated from official government sources for seven countries (USA, UK, Italy, Germany, Spain, France and Korea) [48]. A total of 42 864 confirmed paediatric cases (0-19 years) were reported in these seven countries up to 19/05/2020, and 26.4% of them were under 10 years old. Using updated data from 22 government resources (Appendix S2 of the **Online Supplementary Document**) and data from 7 studies [9,29-34], we report the proportion of children among the confirmed COVID-19 patients for 29 countries, which varies from 0.3% (lowest in Spain) up to 13.8% (highest in Argentina), as shown in **Figure 2**.

### Paediatric Inflammatory Multisystem Syndrome Temporally associated with SARS-CoV-2 (PIMS-TS)

A new paediatric multisystem inflammatory syndrome has been identified and temporally associated with COVID-19. Recent reports from Europe and North America have described a small number of children being admitted to intensive care units with PIMS-TS having some features similar to Kawasaki disease and toxic shock syndrome. This is rare but with common characteristics that have been observed during the COVID-19 pandemic reported from different cohorts [22-27]. A retrospective, observational study performed at 4 academic tertiary care centres in Paris reported a case series of acute myocarditis and major systemic inflammation following SARS-CoV-2 infection in 20 critically ill children [28]. The Royal College of Paediatrics and Child Health estimated that there were approximately 200 cases of PIMS-TS associated with SARS-CoV-2 in the UK among children under 18 years by early June. In spite of the severity of PIMS-TS, there was one death in the cohort of 58 cases in an Imperial College study [25].

## DISCUSSION

This updated rapid evidence review summarises the most recently available evidence to understand the role of children in SARS-CoV-2 transmission. There is a lack of direct evidence on the dynamics of child transmission, however the evidence to date suggests that children are unlikely to be major transmitters of SARS-CoV-2.

Direct evidence showing children as a source of transmission is scarce and largely based on small studies or studies investigating few paediatric cases. We did not identify more studies that reported documented cases of SARS-CoV-2 transmission by children. The original two studies included in our last rapid review [3] indicated that children can transmit COVID-19 to other children or adults [35,36], however, these studies have a high degree of uncertainty because of the difficulty in tracking transmission chains. From this update, we included studies that demonstrated the children’s susceptibility and transmission risk of SARS-CoV-2 with providing evidence on the chance of being index and secondary cases, the potential of faeco-oral transmission, and the possibility of asymptomatic transmission.

By synthesizing preliminary evidence in this update, our study shows that children have lower susceptibility for SARS-CoV-2 infection. A meta-analysis of eight studies reporting an OR for secondary attack ratios of children (vs adults) indicated that children were perhaps 50% less susceptible to infection than adults (odds ratio (OR) = 0.44, 95% CI: 0.29-0.69), with substantial heterogeneity [14]. Of them, six studies found a significant difference in the secondary attack risk between children and adults [7,9-13], while two studies in China, of Bi et al and Jing et al [5,8], found no significant difference, reporting an OR of 0.90 (95% CI = 0.52-1.57) and 0.57 (95% CI = 0.30-1.11). In spite of the heterogeneity between studies, the effect estimates point to the same direction (OR<1), consistently showing a lower infection risk in children. Furthermore, evidence suggests children are infrequently index cases. We found one case report in our last rapid review showing that an infected infant contaminated (with SARS-CoV-2) the surfaces they were in contact with, thus rendering the risk of transmitting the virus to caregivers through indirect contact with fomites [38]. For this update, we found that children were rarely the first person to bring the infection into households, and were responsible for only around 5%-10% of clusters, indicating children were not the main transmission source in household infections [4,5]. This may be due to school closures occurring in most locations along with or before the widespread of COVID-19, therefore, most close contacts became limited to households, reducing the opportunities for children to become infected in the community and present as index cases.

The persistent shedding of SARS-CoV-2 in stools of infected children were continually reported by new studies, which is in accordance with our findings from three studies in our last update, showing that SARS-CoV-2 may be present in the gastrointestinal tract for a longer duration than viral presence in the respiratory system [39-41]. Therefore, much attention has been focused on the possibility of faeco-oral transmission. However, there has not been any documentation of any subsequent infection in family contacts of faecal viral excreting children [15]. The relative infectiousness and viral load of children vs adults is still uncertain. Available evidence showed that young children (<10 years old) had statistically significant lower viral load than adults [49], and symptomatic children had higher initial viral load in nasopharyngeal swab specimens than asymptomatic children [16,17]. The findings corroborated the prior studies demonstrating correlation between viral load and disease severity in younger children [42,49]. It is hypothesized that children with a lower viral load may release fewer infectious particles into the surrounding environment and are thus less infectious to others.

There are very few data sets available on school specific transmission. Although a few outbreaks have been observed in schools, eg, in France (Oise), Australia (New South Wales) and Israel, it is not certain whether the source was from children or adults [21,43] (for more transmission contact-tracing details please see our last rapid review). The new data from Ireland, Belgium, Singapore and the Netherlands shows that the extent of any student-to-student or student-to-teacher/school staff spread is still limited [18-20]. We cannot rule out the possibility that the small number of school outbreaks reported is due to the early school closure in many countries during the epidemic. As many countries have started re-opening schools, monitoring school re-opening and assessing the number of new COVID-19 cases related to schools may give some insight into whether school settings have any impact on the number of COVID-19 infections at a national level.

Some countries (Iceland, Italy, South Korea, Netherlands, and US) have implemented seroprevalence surveys. Data from these countries confirm that the percentage of children among the confirmed COVID-19 patients is small, varying from 1% in young children up to 6% in older children [3]. In addition, we identified two new surveys from Spain and Sweden [45,46]. Both countries found children were significantly underrepresented. The seroprevalence of COVID-19 was reported to be 3%-4% of children tested, comparing to 5.0% in overall population, and the corresponding figure in Sweden was 4.9%, compared to 6.7% of those aged 20-64 years adults. Although the Spanish seroprevalence survey tested a large number of individuals (n = 61 075), the number of children tested (n = 6275) was in a relatively small fraction, and was less than the overall children population in Spain (18%-20%). This likely reflects lower recruitment of children and may be a source of bias. It is possible that biases in population selection for testing or false-negative swabs due to the difficulty in obtaining a sample in children may contribute to these findings. Overall, we should interpret these seroprevalence studies carefully because they are limited by the small numbers of children tested and the potential of biased non-random sampling. The significantly lower seroprevalence in children does point to a comparatively low secondary attack-rate for household contacts where the secondary case is a child as mentioned above.

Beyond the seroprevalence, we also estimated the percentage of children among the confirmed COVID-19 patients based on data from governmental websites and publications. Data for 29 countries shows a substantial varying range (0.3%-13.8%) of proportion of paediatric patients. Although we have tried to find the most recent and comprehensive data for as many countries as possible, data for paediatric patients are not always available for most countries, or have not been recently updated on their governmental websites. We supplemented the governmental data by extracting relevant information from the publications, however, some of them are now out of date at the time of writing. We need to acknowledge that the reported figures may change along with progress of the pandemic in reported countries.

It is widely acknowledged that children are less likely to suffer severe and critical COVID-19 disease than adults [50]; however, PIMS-TS has been reported and hence generated significant media attention and concerns among parents. Although the identified publications suggest a link between PIMS-TS and COVID-19 disease [22-28], it is crucial to be aware that children remain minimally affected by SARS-CoV-2 infection overall according to the national data from Italy, France and UK. Understanding this inflammatory condition might provide important information about immune responses to SARS-CoV-2 in children and it might also help to explain why some children become very ill with SARS-CoV-2 infection, while the majority are unaffected or asymptomatic.

This rapid review has a number of limitations. Despite experienced reviewers undertaking searches, screening and data extraction, due to the tight deadline the literature review and data extraction were done by one person per article and so it is possible that some key articles may have been missed. We have not performed risk of bias assessment for the included studies and so this may have biased the results. Many of the included studies are pre-print publications or reports and therefore not peer-reviewed. The views expressed represent those of the authors and are not a substitute for professional medical advice.

In conclusion, although there is limited evidence available for quantifying the extent to which children may contribute to overall (nationwide) transmission, given the lower susceptibility, relatively small proportion of infections and lower chance of being index cases, children are unlikely to have been significant drivers of the epidemic so far and there is not any evidence yet to indicate they are at higher risk of causing super-spreading events in the community or schools. The balance of evidence suggests that children play only a limited role in overall transmission, but it is noted that the relative contribution of children to SARS-CoV-2 transmission may change with reopening of society and schools.

## Additional material

Online Supplementary Document

## References

[1] Levin M Childhood Multisystem Inflammatory Syndrome - A New Challenge in the Pandemic. N Engl J Med. 2020;383:393-5. 10.1056/NEJMe2023158 32598829 PMC7346677

[2] Royal Society DELVE Initiative. Balancing the Risks of Pupils Returning to Schools. Available: https://rs-delve.github.io/reports/2020/07/24/balancing-the-risk-of-pupils-returning-to-schools.html#fn:49. Accessed: 25 July 2020.

[3] Li X Xu W Dozier M He Y Kirolos A Theodoratou E The role of children in transmission of SARS-CoV-2: A rapid review. J Glob Health. 2020;10:011101. 10.7189/jogh.10.011101 32612817 PMC7323934

[4] Zhu Y, Bloxham CJ, Hulme KD, Sinclair JE, Tong ZWM, Steele LE, et al. Children are unlikely to have been the primary source of household SARS-CoV-2 infections. medRxiv. 2020. Available: https://www.medrxiv.org/content/10.1101/2020.03.26.20044826v1. Accessed: 25 July 2020.

[5] Jing Q-L, Liu M-J, Yuan J, Zhang Z-B, Zhang A-R, Dean NE, et al. Household Secondary Attack Rate of COVID-19 and Associated Determinants. medRxiv. 2020. Available: https://www.medrxiv.org/content/10.1101/2020.04.11.20056010v1. Accessed: 25 July 2020. 10.1016/S1473-3099(20)30471-0 PMC7529929 32562601

[6] National Institute for Public Health and the Environment. Initial results on how COVID-19 spreads within Dutch families. Available: https://www.rivm.nl/en/news/initial-results-on-how-covid-19-spreads-within-dutch-families. Accessed: 25 July 2020.

[7] Zhang J Litvinova M Liang Y Wang Y Wang W Zhao S Changes in contact patterns shape the dynamics of the COVID-19 outbreak in China. Science. 2020;368:1481-6. 10.1126/science.abb8001 32350060 PMC7199529

[8] Bi Q Wu Y Mei S Ye C Zou X Zhang Z Epidemiology and transmission of COVID-19 in 391 cases and 1286 of their close contacts in Shenzhen, China: a retrospective cohort study. Lancet Infect Dis. 2020;20:911-9. 10.1016/S1473-3099(20)30287-5 32353347 PMC7185944

[9] Wu J Huang Y Tu C Bi C Chen Z Luo L Household Transmission of SARS-CoV-2, Zhuhai, China, 2020. Clin Infect Dis. 2020.Online ahead of print. 10.1093/cid/ciaa557 32392331 PMC7239243

[10] Wang Z Ma W Zheng X Wu G Zhang R Household transmission of SARS-CoV-2. J Infect. 2020;81:179-82. 10.1016/j.jinf.2020.03.040 32283139 PMC7151261

[11] Li W Zhang B Lu J Liu S Chang Z Cao P The characteristics of household transmission of COVID-19. Clin Infect Dis. 2020. Online ahead of print. 10.1093/cid/ciaa450 32301964 PMC7184465

[12] Cheng HY Jian SW Liu DP Ng TC Huang WT Lin HH Contact Tracing Assessment of COVID-19 Transmission Dynamics in Taiwan and Risk at Different Exposure Periods Before and After Symptom Onset. JAMA Intern Med. 2020;180:1156-63. 10.1001/jamainternmed.2020.2020 32356867 PMC7195694

[13] Mizumoto K Kagaya K Zarebski A Chowell G Estimating the asymptomatic proportion of coronavirus disease 2019 (COVID-19) cases on board the Diamond Princess cruise ship, Yokohama, Japan, 2020. Euro Surveill. 2020;25:2000180. 10.2807/1560-7917.ES.2020.25.10.2000180 32183930 PMC7078829

[14] Viner RM, Mytton OT, Bonell C, Melendez-Torres GJ, Ward JL, Hudson L, et al. Susceptibility to and transmission of COVID-19 amongst children and adolescents compared with adults: a systematic review and meta-analysis. medRxiv. 2020. Available: https://www.medrxiv.org/content/10.1101/2020.05.20.20108126v2. Accessed: 25 July 2020.

[15] Hua CZ Miao ZP Zheng JS Huang Q Sun QF Lu HP Epidemiological features and viral shedding in children with SARS-CoV-2 infection. J Med Virol. 2020. Online ahead of print. 10.1002/jmv.26180 32542750 PMC7323101

[16] Han MS Seong MW Kim N Shin S Cho SI Park H Viral RNA Load in Mildly Symptomatic and Asymptomatic Children with COVID-19, Seoul. Emerg Infect Dis. 2020;26:2497-9. 10.3201/eid2610.202449 32497001 PMC7510743

[17] Pandey U, Yee R, Precit M, Bootwalla M, Ryutov A, Shen L, et al. Pediatric COVID-19 in Southern California: clinical features and viral genetic diversity. medRxiv. 2020. Available: https://www.medrxiv.org/content/10.1101/2020.05.28.20104539v2. Accessed: 25 July 2020.

[18] Desmet S, Ekinci E, Wouters I, Decru B, Beuselinck K, Malhotra-Kumar S, et al. No SARS-CoV-2 carriage observed in children attending daycare centers during the first weeks of the epidemic in Belgium. medRxiv. 2020. Available: https://www.medrxiv.org/content/10.1101/2020.05.13.20095190v1. Accessed: 25 July 2020. 10.1002/jmv.26689 PMC7753838 33230857

[19] Heavey L Casey G Kelly C Kelly D McDarby G No evidence of secondary transmission of COVID-19 from children attending school in Ireland, 2020. Euro Surveill. 2020;25:2000903. 10.2807/1560-7917.ES.2020.25.21.2000903 32489179 PMC7268273

[20] Yung CF Kam KQ Nadua KD Chong CY Tan NWH Li J Novel coronavirus 2019 transmission risk in educational settings. Clin Infect Dis. 2020. Online ahead of print. 10.1093/cid/ciaa794 32584975 PMC7337629

[21] Clalit Health Services. Corona Patients In Israel: Situation Clalit Health Services. 2020. Available: https://www.clalit.co.il/he/your_health/family/Pages/corona_in_israel.aspx. Accessed: 19 July 2020.

[22] Viner RM Whittaker E Kawasaki-like disease: emerging complication during the COVID-19 pandemic. Lancet. 2020;395:1741-3. 10.1016/S0140-6736(20)31129-6 32410759 PMC7220168

[23] Riphagen S Gomez X Gonzalez-Martinez C Wilkinson N Theocharis P Hyperinflammatory shock in children during COVID-19 pandemic. Lancet. 2020;395:1607-8. 10.1016/S0140-6736(20)31094-1 32386565 PMC7204765

[24] Jones VG Mills M Suarez D Hogan CA Yeh D Segal JB COVID-19 and Kawasaki Disease: Novel Virus and Novel Case. Hosp Pediatr. 2020;10:537-40. 10.1542/hpeds.2020-0123 32265235

[25] Whittaker E Bamford A Kenny J Kaforou M Jones CE Shah P Clinical Characteristics of 58 Children With a Pediatric Inflammatory Multisystem Syndrome Temporally Associated With SARS-CoV-2. JAMA. 2020;324:259-69. 10.1001/jama.2020.10369 32511692 PMC7281356

[26] Verdoni L Mazza A Gervasoni A Martelli L Ruggeri M Ciuffreda M An outbreak of severe Kawasaki-like disease at the Italian epicentre of the SARS-CoV-2 epidemic: an observational cohort study. Lancet. 2020;395:1771-8. 10.1016/S0140-6736(20)31103-X 32410760 PMC7220177

[27] Belhadjer Z Méot M Bajolle F Khraiche D Legendre A Abakka S Acute heart failure in multisystem inflammatory syndrome in children (MIS-C) in the context of global SARS-CoV-2 pandemic. Circulation. 2020. Online ahead of print. 10.1161/CIRCULATIONAHA.120.048360 32418446

[28] Grimaud M Starck J Levy M Marais C Chareyre J Khraiche D Acute myocarditis and multisystem inflammatory emerging disease following SARS-CoV-2 infection in critically ill children. Ann Intensive Care. 2020;10:69. 10.1186/s13613-020-00690-8 32488505 PMC7266128

[29] Levy C, Basmaci R, Bensaid P, Bru C, Coinde E, Dessioux E, et al. Changes in RT-PCR-positive SARS-CoV-2 rates in adults and children according to the epidemic stages 2020. Available: https://www.medrxiv.org/content/10.1101/2020.05.18.20098863v2. Accessed: 19 July 2020. 10.1097/INF.0000000000002861 32868745

[30] Nikpouraghdam M Jalali Farahani A Alishiri G Heydari S Ebrahimnia M Samadinia H Epidemiological characteristics of coronavirus disease 2019 (COVID-19) patients in IRAN: A single center study. J Clin Virol. 2020;127:104378. 10.1016/j.jcv.2020.104378 32353762 PMC7172806

[31] Mizumoto K, Omori R, Nishiura H. Age specificity of cases and attack rate of novel coronavirus disease (COVID-19). medRxiv. 2020. Available: https://www.medrxiv.org/content/10.1101/2020.03.09.20033142v1. Accessed: 19 July 2020.

[32] Kemelbekov K Ospanova E Baimakhanova B Zhumabekov Z Zholdas K Yessentayeva Z Epidemiological Characteristics of New Coronavirus Diseases (COVID-19): Features of Risk Factors and Clinical Features of the Child Population. Electron J Gen Med. 2020;17:em252 10.29333/ejgm/8268

[33] Choe YJ. Coronavirus disease-19: The First 7,755 Cases in the Republic of Korea. medRxiv. 2020. Available: https://www.medrxiv.org/content/10.1101/2020.03.15.20036368v1. Accessed: 19 July 2020.

[34] National Institute for Communicable Diseases. South Africa-2020. COVID-19 update. Available: https://www.nicd.ac.za/covid-19-update-46/. Accessed: 15 July 2020.

[35] Jiehao C Jin X Daojiong L Zhi Y Lei X Zhenghai Q A Case Series of children with 2019 novel coronavirus infection: clinical and epidemiological features. Clin Infect Dis. 2020;71:1547-51. 10.1093/cid/ciaa198 32112072 PMC7108143

[36] National Centre for Immunisation Research and Surveillance (NCIRS). COVID-19 in schools – the experience in NSW. Available: http://ncirs.org.au/sites/default/files/2020-04/NCIRS%20NSW%20Schools%20COVID_Summary_FINAL%20public_26%20April%202020.pdf. Accessed: 26 April 2020.

[37] Dong Y Mo X Hu Y Qi X Jiang F Jiang Z Epidemiology of COVID-19 Among Children in China. Pediatrics. 2020;145:e20200702. 10.1542/peds.2020-0702 32179660

[38] Yung CF Kam KQ Wong MSY Maiwald M Tan YK Tan BH Environment and Personal Protective Equipment Tests for SARS-CoV-2 in the Isolation Room of an Infant With Infection. Ann Intern Med. 2020;173:240-2. 10.7326/M20-0942 32236490 PMC7133054

[39] Xu Y Li X Zhu B Liang H Fang C Gong Y Characteristics of pediatric SARS-CoV-2 infection and potential evidence for persistent fecal viral shedding. Nat Med. 2020;26:502-5. 10.1038/s41591-020-0817-4 32284613 PMC7095102

[40] Ma X Su L Zhang Y Zhang X Gai Z Zhang Z Do children need a longer time to shed SARS-CoV-2 in stool than adults? J Microbiol Immunol Infect. 2020;53:373-6. 10.1016/j.jmii.2020.03.010 32224116 PMC7138153

[41] Xing YH Ni W Wu Q Li WJ Li GJ Wang WD Prolonged viral shedding in feces of pediatric patients with coronavirus disease 2019. J Microbiol Immunol Infect. 2020;53:473-80. 10.1016/j.jmii.2020.03.021 32276848 PMC7141453

[42] Terry C, Barbara M, Talitha V, Marta Z, Jorg H, Angela S, et al. An analysis of SARS-CoV-2 viral load by patient age. Available: https://zoonosen.charite.de/fileadmin/user_upload/microsites/m_cc05/virologie-ccm/dateien_upload/Weitere_Dateien/analysis-of-SARS-CoV-2-viral-load-by-patient-age.pdf. Accessed: 6 May 2020.

[43] Fontanet A, Tondeur L, Madec Y, Grant R, Besombes C, Jolly N, et al. Cluster of COVID-19 in northern France: A retrospective closed cohort study. medRxiv. 2020. Available: https://www.medrxiv.org/content/10.1101/2020.04.18.20071134v1. Accessed: 6 October 2020.

[44] Danis K Epaulard O Bénet T Gaymard A Campoy S Bothelo-Nevers E Cluster of coronavirus disease 2019 (Covid-19) in the French Alps, 2020. Clin Infect Dis. 2020;71:825-32. 10.1093/cid/ciaa424 32277759 PMC7184384

[45] Pollán M Pérez-Gómez B Pastor-Barriuso R Oteo J Hernán MA Pérez-Olmeda M Prevalence of SARS-CoV-2 in Spain (ENE-COVID): a nationwide, population-based seroepidemiological study. Lancet. 2020;396:535-44. 10.1016/S0140-6736(20)31483-5 32645347 PMC7336131

[46] Public Health Agency of Sweden. Initial results from ongoing investigation of antibodies to COVID-19 virus, 20 May 2020.Available: https://www.folkhalsomyndigheten.se/nyheter-och-press/nyhetsarkiv/2020/maj/forsta-resultaten-fran-pagaende-undersokning-av-antikroppar-for-COVID-19-virus/. Accessed: 15 July 2020*.*

[47] Barton Forbes M, Mehta K, Kumar K, Lu J, Le Saux N, Sampson M, et al. COVID- 19 Infection in Children: Estimating Pediatric Morbidity and Mortality. medRxiv. 2020. Available: https://www.medrxiv.org/content/10.1101/2020.05.05.20091751v1. Accessed: 6 October 2020.

[48] Bhopal S Bagaria J Bhopal R Children’s mortality from COVID-19 compared with all-deaths and other relevant causes of death: epidemiological information for decision-making by parents, teachers, clinicians and policymakers. Public Health. 2020;185:19-20. 10.1016/j.puhe.2020.05.047 32516623 PMC7260492

[49] Is SARS-CoV-2 viral load lower in young children than adults? Available: https://medium.com/@d_spiegel/is-sars-cov-2-viral-load-lower-in-young-children-than-adults-8b4116d28353. Accessed: 26 May 2020.

[50] Ludvigsson JF Systematic review of COVID-19 in children shows milder cases and a better prognosis than adults. Acta Paediatr. 2020;109:1088-95. 10.1111/apa.15270 32202343 PMC7228328

