# Supplementary material for: The role of children in the transmission of SARS-CoV2: updated rapid review
Source: J Glob Health. 2020 Sep 23;10(2):021101. doi: 10.7189/jogh.10.021101 (PMC7719356; doi:10.7189/jogh.10.021101)
Supplement: Online Supplementary Document [file jogh-10-021101-s001.pdf]

Appendix

Search Strategy:

PubMed

Date of search: 2020-06-21

Schools[mh:noexp] OR schools, nursery[mh] OR “Child Day Care Centers”[mh] OR “Nurseries, Infant”[mh] OR school[tiab] OR schools[tiab] OR preschools[tiab] OR preschool[tiab] OR “pre school”[tiab] OR “pre schools”[tiab] OR nursery[tiab] OR nurseries[tiab] OR kindergarten[tiab] OR ((“day care” OR daycare) AND (child* OR infant*)) OR pediatric[tiab] OR paediatric[tiab] OR child[tiab] or children[tiab] or schoolchild*[tiab] OR young*[tiab] OR teen*[tiab] OR adolescen*

AND

"Betacoronavirus"[Mesh] OR "Coronavirus Infections"[MH] OR "Spike Glycoprotein, COVID-19 Virus”[NM] OR "COVID-19"[NM] OR "Coronavirus"[MH] OR "Severe Acute Respiratory Syndrome Coronavirus 2"[NM] OR 2019nCoV[ALL] OR Betacoronavirus*[ALL] OR Corona Virus*[ALL] OR Coronavirus*[ALL] OR Coronovirus*[ALL] OR CoV[ALL] OR CoV2[ALL] OR COVID[ALL] OR COVID19[ALL] OR COVID-19[ALL] OR HCoV-19[ALL] OR nCoV[ALL] OR "SARS CoV 2"[ALL] OR SARS2[ALL] OR SARSCoV[ALL] OR SARS-CoV[ALL] OR SARS-CoV-2[ALL] OR Severe Acute Respiratory Syndrome CoV*[ALL]

WHO COVID-19 database https://search.bvsalud.org/global-literature-on-novel-coronavirus-2019-ncov/

Date of search: 2020-06-21

(tw:(child* OR infan* OR baby OR babies OR pediatric OR paediatric OR young* OR teen* OR adolescen* OR school* OR nurser* OR preschool* OR pre-school* OR kindergarten*)) AND (entry_date:202004*)

medRxiv via https://mcguinlu.shinyapps.io/medrxivr/

Date of search: 2020-06-21

Covid-19 cluster combined with OR:

COVID-19

[Cc]oronavirus

SARS-CoV-2

2019-nCoV

Population cluster combined with OR:

[Cc]hild

[Ii]nfan

[Bb]aby

[Bb]abies

[Pp]ediatric

[Pp]aediatric

[Yy]oung

[Tt]een

[Aa]dolescen

[Ss]chool

[Nn]urser

[Pp]reschool

[Pp]re-school

[Kk]indergarten

Supplementary Table S1. Data sources reporting the proportion of childhood infection of COVID.

Country	Data source	No of COVID cases	No of pediatric cases	Proportion of childhood infection	Data link
Algeria	Website	17348	429	0.0247	http://covid19.cipalgerie.com/fr/statistiques-detaillees-covid-19-algerie/
Argentina	Website	87017	11983	0.1377	https://www.argentina.gob.ar/salud/coronavirus-COVID-19/sala-situacion
Australia	Report	5805	190	0.0327	https://www1.health.gov.au/internet/main/publishing.nsf/Content/1D03BCB527F40C8BCA258503000302EB/$File/covid_19_australia_epidemiology_report_10__reporting_week_ending_23_59_aest_5_april_2020.pdf
Austria	Website	18548	778	0.0419	https://info.gesundheitsministerium.at/dashboard_Epidem.html?l=de
Canada	Report	24804	721	0.0291	https://www.canada.ca/content/dam/phac-aspc/documents/services/diseases/2019-novel-coronavirus-infection/surv-covid19-epi-update-2020-04-13-eng.pdf
China	Population-based	44672	965	0.0216	Wu-2020 (DOI: 10.1093/cid/ciaa557)
Colombia	Website	2852	178	0.0624	https://www.ins.gov.co/Noticias/Paginas/Coronavirus.aspx
Denmark	Report	6496	236	0.0363	https://files.ssi.dk/COVID19-overvaagningsrapport-14042020-wgkv
Ecuador	Report	7466	379	0.0508	https://www.salud.gob.ec/wp-content/uploads/2020/04/Boletin-045-AM_Nacional.pdf
Estonia	Website	1332	47	0.0353	https://www.terviseamet.ee/et/koroonaviirus/koroonakaart
Finland	Website	3064	204	0.0666	https://thl.fi/en/web/infectious-diseases-and-vaccinations/what-s-new/coronavirus-covid-19-latest-updates
France	Hospital-based	9728	382	0.0393	Levy, C.-2020 (DOI: 10.1101/2020.05.18.20098863)
Germany	Website	197783	8378	0.0424	https://experience.arcgis.com/experience/478220a4c454480e823b17327b2bf1d4/page/page_0/
Greece	Report	743	27	0.0363	https://eody.gov.gr/wp-content/uploads/2020/03/covid-gr-daily-report-20200324.pdf
Iceland	Website	1810	180	0.0994	https://www.covid.is/data
Iran	Hospital-based	2964	10	0.0034	Nikpouraghdam, M.-2020 (DOI: 10.1016/j.jcv.2020.104378)
Ireland	Website	9484	115	0.0121	https://www.gov.ie/en/publication/b86091-an-analysis-of-the-836-cases-of-covid-19-in-ireland-as-of-saturday-2/#age-range-affected
Japan	Population-based	294	10	0.0340	Mizumoto-2020 (DOI: 10.1101/2020.03.09.20033142)
Kazakhastan	Population-based study	1295	80	0.0618	Kemelbekov, K.-2020 (https://doi.org/10.29333/ejgm/8268)
Netherlands	Website	27419	410	0.0150	https://www.rivm.nl/coronavirus-covid-19/grafieken
New Zealand	Website	1540	159	0.1032	https://www.health.govt.nz/our-work/diseases-and-conditions/covid-19-novel-coronavirus/covid-19-current-situation/covid-19-current-cases
Norway	Website	6623	353	0.0533	https://www.vg.no/spesial/2020/corona/
Pakistan	Website	5496	374	0.0680	http://covid.gov.pk/stats/pakistan
Republic of Korea	Population-based	7755	480	0.0619	Choe-2020 (DOI: 10.1101/2020.03.15.20036368)
Republic of Moldova	Website	18471	1207	0.0653	http://gismoldova.maps.arcgis.com/apps/opsdashboard/index.html#/d274da857ed345efa66e1fbc959b021b

South Africa	Population-based	3144	134	0.0426	National Institute for Communicable Diseases, South Africa-2020 (https://www.nicd.ac.za/covid-19-update-46/)
Spain	Website	135032	349	0.0026	https://www.isciii.es/QueHacemos/Servicios/VigilanciaSaludPublicaRENAVE/EnfermedadesTransmisibles/Documents/INFORMES/Informes%20COVID-19/Informe%20n%C2%BA%2021.%20Situaci%C3%B3n%20de%20COVID-19%20en%20Espa%C3%B1a%20a%206%20de%20abril%20de%202020.pdf
Sweden	Website	74072	3316	0.0448	https://platz.se/coronavirus/
Switzerland	Website	32341	1239	0.0383	https://covid-19-schweiz.bagapps.ch/fr-1.html
